# MoS_2_-Based Composites for Electrochemical Detection of Heavy Metal Ions: A Review

**DOI:** 10.3390/nano15211639

**Published:** 2025-10-27

**Authors:** Baizun Cheng, Hongdan Wang, Shouqin Xiang, Shun Lu, Bingzhi Ren

**Affiliations:** 1 School of Metallurgy and Materials Engineering, Chongqing University of Science and Technology, Chongqing 401331, China; 2 Chongqing Institute of Green and Intelligent Technology, Chinese Academy of Sciences, Chongqing 400714, China

**Keywords:** MoS_2_, heavy metal ions, electrochemical detection, nanocomposites

## Abstract

Heavy metal ions (HMIs) threaten ecosystems and human health due to their carcinogenicity, bioaccumulativity, and persistence, demanding highly sensitive, low-cost real-time detection. Electrochemical sensing technology has gained significant attention owing to its rapid response, high sensitivity, and low cost. Molybdenum disulfide (MoS_2_), with its layered structure, tunable bandgap, and abundant edge active sites, demonstrates significant potential in the electrochemical detection of heavy metals. This review systematically summarizes the crystal structure characteristics of MoS_2_, various preparation strategies, and their mechanisms for regulating electrochemical sensing performance. It particularly explores the cooperative effects of MoS_2_ composites with other materials, which effectively enhance the sensitivity, selectivity, and detection limits of electrochemical sensors. Although MoS_2_-based materials have made significant progress in theoretical and applied research, practical challenges remain, including fabrication process optimization, interference from complex-matrix ions, slow trace-metal enrichment kinetics, and stability issues in flexible devices. Future work should focus on developing efficient, low-cost synthesis methods, enhancing interference resistance through microfluidic and biomimetic recognition technologies, optimizing composite designs, resolving interfacial reaction dynamics via in situ characterization, and establishing structure–property relationship models using machine learning, ultimately promoting practical applications in environmental monitoring, food safety, and biomedical fields.

## 1. Introduction

Heavy metals are naturally present in the Earth’s crust. In recent years, with the continuous growth of the population, excessive exploitation of heavy metals, expansion of the metal smelting industry, and the discharge of agricultural and domestic waste, the content of heavy metals in the environment has significantly increased [1]. Heavy metal pollution primarily originates from wastewater generated by industrial and domestic pollution. Industrial pollution: Most industrial production activities are accompanied by the generation of heavy metal pollutants, with mining and smelting being the main sources of heavy metal pollution. During the mining process, scattered open-pit tailings can be washed away by wind and rain, infiltrating groundwater and subsequently contaminating water sources [2]. Another important source is the chemical industry, which generates a large amount of industrial wastewater containing heavy metals in various industries such as electroplating, batteries, fertilizers, tannery, textile printing and dyeing, and paints. Domestic pollution: In our daily lives, the various industrial products we use, such as preservatives, detergents, cosmetics, electronic products, and paints, all contain certain amounts of heavy metals. When these are discharged or disposed of directly into the environment without treatment, they enter groundwater and soil, causing pollution [3,4].

The exponential growth of HMIs poses a serious threat to the health of various biological species through bioaccumulation in the food chain. When the human body ingests these heavy metals via food, water, or other means, it may lead to symptoms such as forgetfulness, insomnia, joint pain, dizziness, and nausea, and may even cause various cancers [5]. Heavy metals refer to metals with an atomic relative density greater than 4.5 g/cm^3^. Common HMIs include iron, zinc, cobalt, cadmium, copper, and manganese. Among these, iron, zinc, copper, and manganese are essential trace elements for the human body; however, when their concentrations exceed a certain threshold, they may have adverse effects on health. Lead, copper, mercury, and cadmium are the most frequently detected heavy metal pollutants in the environment, largely due to their non-biodegradable nature. These heavy metals can accumulate in organs through bioaccumulation, posing a significant threat to human health and substantially increasing the risk of cancer [6,7]. Therefore, the development of more convenient, sensitive, selective, and cost-effective heavy metal detection technologies holds significant importance for effectively preventing heavy metal pollution and ensuring human health.

Current analytical methods for HMIs are diverse and can be primarily classified by detection principles into the following categories: atomic spectroscopy [8], mass spectrometry [9], inductively coupled plasma mass spectrometry [10], spectrophotometry [11], biosensor techniques [12], and electrochemical methods [13]. Compared with other analytical methods, electrochemical techniques exhibit high sensitivity, low detection limits, rapid analysis speed, and broad measurement ranges, enabling the simultaneous determination of multiple metal species. They are characterized by simple instrumentation, low cost, high portability, and capabilities for miniaturization and integration, which support on-site detection and real-time monitoring [14]. Furthermore, studies demonstrate that integrating diverse electrochemical techniques with advanced sensing strategies, along with utilizing modified nanomaterials on electrode surfaces, significantly enhances sensor sensitivity and minimizes detection limits [15,16].

Among the materials used for electrode modification, 2D materials have shown tremendous potential. Transition metal dichalcogenides (TMDs) have garnered significant attention due to their unique electrocatalytic, electronic, and optical properties. Their large specific surface area and ultra-thin layered structure make them highly significant in both fundamental research and technological applications. TMDs are molecules where a transition metal layer is sandwiched between two chalcogen elements, forming an MX_2_ structure. Their distinctive physicochemical properties amplify signals and accelerate bio-recognition responses, rendering them applicable in electronic and optical fields [17,18,19]. Currently, the most extensively researched and widely applied TMD is MoS_2_.

**Figure 1:**
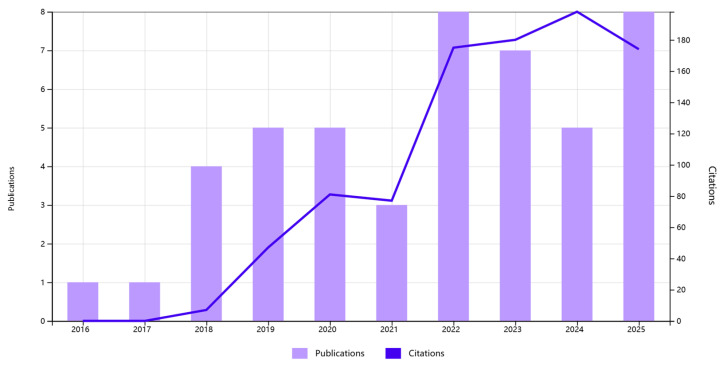
Annual number of publications and citations on MoS_2_-based electrochemical sensors for heavy metal ions.

In recent years, research interest in MoS_2_-based electrochemical sensors for the detection of HMIs has demonstrated remarkable growth. As illustrated in Figure 1, which summarizes the annual number of publications and corresponding citation counts retrieved from the Web of Science (topic: molybdenum disulfide and electrochemical detection and heavy metal ion), this field has experienced a rapid and steady expansion since 2016. It can be seen that both the annual publication volume and the total number of citations have maintained rapid growth. This clear growth trend not only confirms the enormous potential of MoS_2_ materials in this application field, which is widely recognized, but also highlights the importance and necessity of continuous in-depth research on it. This review summarizes recent advances on MoS_2_-based electrode materials for electrochemical detection, with a primary focus on advances in their application within electrochemical sensors. The performance characteristics of MoS_2_ electrodes fabricated via diverse synthesis methods and composite structures are systematically summarized. Key advantages and existing challenges in electrochemical detection are analyzed, along with an outlook on their promising potential for future high-sensitivity and high-selectivity electrochemical sensors.

## 2. Overview of MoS_2_ Electrode Materials

MoS_2_ is a typical layered TMD, featuring a natural hexagonal layered phase and various non-hexagonal metastable phases. Each monolayer consists of three atomic layers (S-Mo-S) covalently bonded in close configuration, with successive layers stacked via weaker van der Waals interactions at an interlayer spacing of approximately 0.65 nm. Depending on atomic layer arrangement and symmetry, MoS_2_ primarily exhibits three crystalline phases: the metastable 1T (trigonal) phase [20], the semiconducting 2H (hexagonal) phase, and the 3R (rhombohedral) phase.

**Figure 2:**
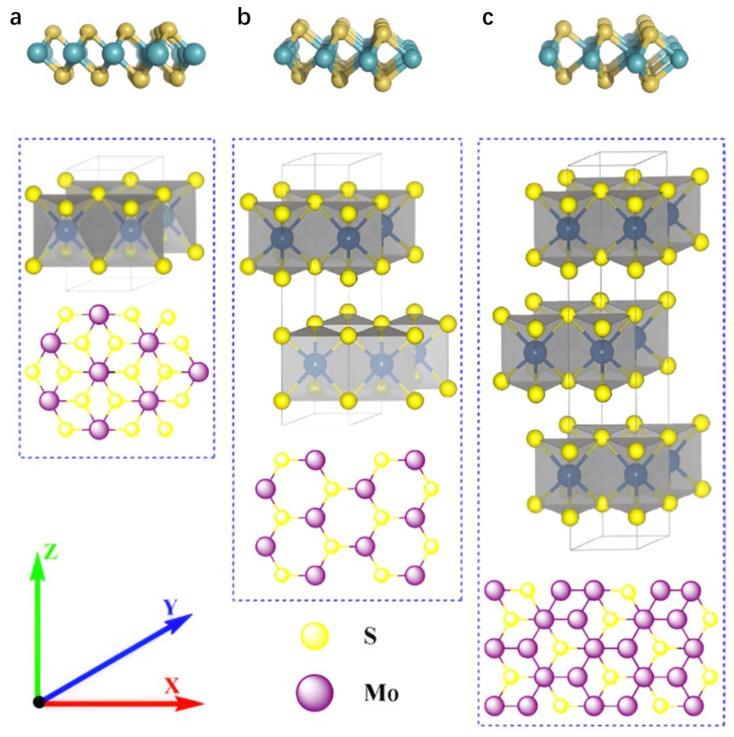
The crystal structure of MoS_2_. (**a**) Octahedral (1T-phase), (**b**) trigonal prismatic (2H-phase) and (**c**) trigonal prismatic (3R-phase) unit cell structures. Reproduced with permission from ref. [21], Copyright 2020, Elsevier.

In Figure 2, the 1T phase exhibits octahedral symmetry, where molybdenum atoms are octahedrally coordinated by six sulfur atoms, forming a metallic structure with minimal bandgap and superior conductivity, making it suitable for electrocatalytic reactions [21]. As the most thermodynamically stable phase in nature, the 2H phase possesses hexagonal symmetry with molybdenum atoms trigonally prism-coordinated to sulfur atoms. This configuration confers semiconducting properties characterized by a larger bandgap (≈1.8–1.9 eV), leading to extensive utilization in optoelectronic devices. The 3R phase features rhombohedral symmetry with a distinct interlayer stacking sequence compared to the 2H phase, while maintaining the trigonal prismatic coordination motif. The distinctions among these phases primarily manifest in unit cell layer numbers and symmetry. Notably, reversible phase transitions between the 1T and 2H phases can be induced by external stimuli (e.g., intercalation or strain) [22,23,24,25]. MoS_2_ nanosheets, as graphene analogues, share similar properties including large specific surface area, ultrathin layer thickness, and exceptional physicochemical, optical, and electrical properties, enabling their broad applications in sensing, lithium-ion batteries, optoelectronics, and energy storage [26,27]. Notably, the crystal phase of MoS_2_ significantly modulates its electrochemical sensing performance. The surface chemical properties and exposed active sites vary with different crystal phases, affecting the interaction with target analytes, which in turn may lead to differences in the selectivity of the sensor for specific analytes and its response to interfering substances. Among them, 1T phase MoS_2_ has a small bandgap. When incorporated into composite materials, its electronic and optical properties undergo significant changes. For example, metallic properties promote electron transfer, enhancing conductivity and electrocatalytic activity [28]. These changes can be monitored through photoelectron spectroscopy to assess electronic structure, or evaluated using spectroscopic techniques to assess optical properties. Additionally, techniques such as high-resolution transmission electron microscopy, X-ray diffraction, and Raman spectroscopy can be employed to map and quantify the distribution and interactions of different phases in MoS_2_-based nanocomposites.

**Figure 3:**
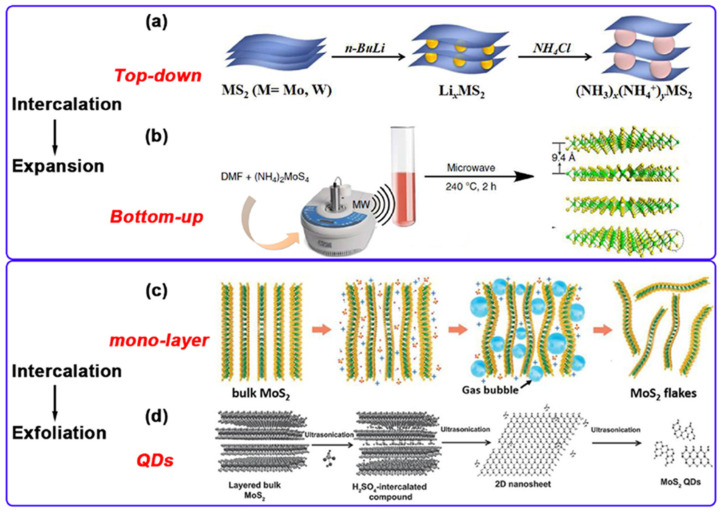
(**a**,**b**): The methods of synthesizing MS_2_ from top to bottom and from bottom to top. (**c**): The intercalation-assisted electrochemical exfoliation of bulk MoS_2_ in 0.5 M Na_2_SO_4_ aqueous solution. (**d**): Intercalation-assistant exfoliation mechanism for preparing MoS_2_ QDs. Reproduced with permission from ref. [29], Copyright 2021, Elsevier.

Currently, diverse methodologies for synthesizing MoS_2_ can be broadly classified into two strategies: top-down and bottom-up approaches. The former involves exfoliating bulk MoS_2_ via physical or chemical means to yield few-layer or monolayer nanosheets, while the latter directly synthesizes MoS_2_ nanostructures through chemical reactions (Figure 3a,b).

### 2.1. Top-Down Approaches

1. Mechanical exfoliation is a classical method for isolating monolayer or few-layer nanosheets from layered materials by applying physical force. This technique exploits the weak interlayer van der Waals forces characteristic of such materials. The standard procedure is as follows: First, a bulk MoS_2_ crystal is positioned onto the surface of a transparent adhesive tape with strong adhesion. Repeated peeling and pressing actions are then employed to mechanically disrupt the interlayer bonding. Subsequently, the tape bearing the exfoliated nanosheets is transferred onto a target substrate (e.g., SiO_2_/Si or gold-coated substrates). Following the slow removal of the tape, the exfoliated nanosheets remain adhered to the substrate surface. Finally, samples with the desired number of layers are identified and selected using optical microscopy or atomic force microscopy [22]. This method was initially demonstrated by Frindt [30] in 1966 for the exfoliation of MoS_2_, producing nanosheets ranging from several to dozens of layers thick. Despite its inherent scaling limitations, mechanical exfoliation remains the preferred method in laboratories for obtaining high-quality two-dimensional (2D) MoS_2_ nanosheets due to the exceptional structural integrity and electronic quality of the resulting samples.
2. Chemical exfoliation is a versatile strategy for the efficient delamination of layered materials. This approach relies on the intercalation of selected chemical agents into the interlayer galleries of MoS_2_. The intercalants induce chemical reactions that expand the interlayer spacing and weaken the van der Waals bonding forces. Subsequent delamination is achieved through sonication or hydrolysis, yielding exfoliated nanosheets with high production yield and robust solution stability [31]. Zheng et al. [32] successfully synthesized monolayer MoS_2_ using sodium naphthalenide (Na^+^C_10_H_8_^−^) and related alkali metal naphthalenide complexes as intercalants via a two-step expansion and intercalation protocol. Their comparative study of lithium, sodium, and potassium-based intercalants revealed that sodium naphthalenide yielded MoS_2_ monolayers with lateral dimensions reaching 400 μm^2^, demonstrating its efficacy for producing high-quality, large-area monolayer MoS_2_. This method offers a straightforward procedure and utilizes reagents with relatively low toxicity. However, the introduced alkali metal ions pose challenges for complete elimination from the final material. Furthermore, certain essential intercalants are costly and present inherent safety hazards during handling.
3. Electrochemical exfoliation represents a method for preparing MoS_2_ nanosheets, as investigated by You et al. [33]. This technique exploits the co-intercalation of SO_4_^2−^ and OH^−^ ions coupled with gas evolution-induced expansion to overcome interlayer van der Waals forces, thereby delaminating bulk MoS_2_ crystals into nanosheets. In their experimental setup, a natural MoS_2_ crystal served as the working electrode, a platinum wire as the counter electrode, and a 0.5 M H_2_SO_4_ aqueous solution as the electrolyte. Application of a bias voltage facilitated the exfoliation process. The resulting MoS_2_ nanosheets exhibited high crystallinity, large lateral dimensions (approximately 20 μm), and a Mo/S atomic ratio approximating 1:2. Liu et al. [34] employed electrochemical exfoliation to synthesize large-area, high-quality MoS_2_ nanosheets. In their procedure, a bulk MoS_2_ crystal served as the working electrode, a platinum wire as the counter electrode, and a 0.5 M sodium sulfate (Na_2_SO_4_) aqueous solution as the electrolyte. Following a +2 V pre-wetting step, a +10 V DC bias voltage was applied to drive the electrochemical exfoliation. This method facilitates the co-intercalation of hydroxyl radicals and sulfate ions, generated in situ via electrolysis, into the MoS_2_ interlayers. The mechanical force exerted by concomitant oxygen gas evolution further promotes interlayer dissociation. Consequently, monolayer and few-layer MoS_2_ nanosheets with lateral dimensions ranging from 5 to 50 μm were successfully obtained (Figure 3c). Material oxidation was effectively suppressed through optimization of the electrolysis parameters. Combining the scalability inherent to solution processing with the high material quality achieved, this approach offers a promising strategy for the controllable exfoliation of TMDs.
4. Liquid-phase exfoliation involves dispersing MoS_2_ powder in water or an organic solvent, followed by ultrasonic treatment to exfoliate it into single or few-layer nanosheets. The method is influenced by factors such as ultrasonic power and solvent choice [35]. Dai et al. [36] were able to gradually exfoliate and cut bulk MoS_2_ into structures of different sizes, including single-layer MoS_2_ flakes, porous MoS_2_ flakes, and quantum dots, by controlling the ultrasonic time (Figure 3d). The experimental results show that the prepared MoS_2_ quantum dots exhibit uniform lateral dimensions of approximately 3.5 nm and a height ranging from 1 to 1.5 nm. They demonstrate excellent excitation-independent blue photoluminescence characteristics, with a quantum yield of 9.65% and a fluorescence lifetime of 4.66 nanoseconds. Furthermore, they exhibit good fluorescence stability within the pH range of 4 to 10. Liquid-phase exfoliation is low-cost and simple to operate, making it suitable for large-scale production. Nevertheless, it has drawbacks such as non-uniform thickness and size of the prepared materials, and difficulty in removing organic solvents.

### 2.2. Bottom-Up Approaches

**Figure 4:**
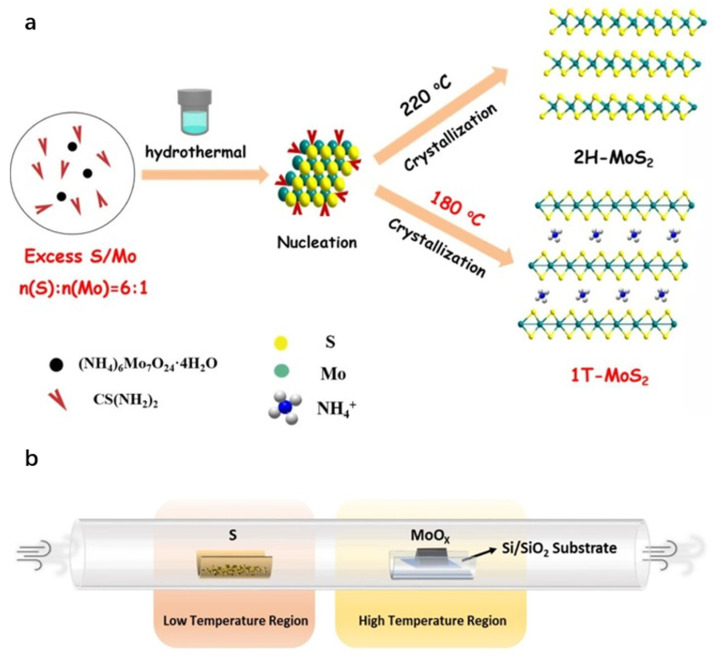
(**a**): Schematic of the hydrothermal method. Reproduced with permission from ref. [38], Copyright 2022, Elsevier. (**b**): Schematic of the chemical vapor deposition method. Reproduced with permission from ref. [43], Copyright 2019, American Chemical Society.

The hydrothermal method is defined as a chemical synthesis technique utilizing water as the solvent within sealed, pressurized autoclaves under elevated temperatures and pressures. It is commonly employed for the synthesis of metal sulfides. The procedure involves dissolving precursors in aqueous solution, sealing the mixture in an autoclave, and heating it to specific temperatures and pressures to facilitate the reaction and formation of the target product. Although this method offers operational simplicity and enables control over product morphology and structure through modulation of reaction conditions and precursor selection, it requires relatively high temperatures and pressures, raising inherent safety concerns. The solvothermal method extends the principles of hydrothermal synthesis by replacing water with organic solvents, thereby providing distinct reaction environments suitable for synthesizing specific materials [37]. Zhang et al. [38] used an NH_3_ intercalation method and a one-pot hydrothermal approach to synthesize ultrathin 1T-MoS_2_ nanosheets. The key to forming 1T-MoS_2_ was using excess thiourea (a sulfur-to-molybdenum molar ratio of 6:1) and a suitable hydrothermal temperature of 180 °C. Under the same conditions except for temperature, 2H-MoS_2_ was synthesized at 220 °C (Figure 4a). Zhai et al. [39] employed water as the solvent, sodium molybdate as the molybdenum source, and thiourea as the sulfur source. Reacting these precursors at 200 °C for 24 h yielded MoS_2_ with a two-dimensional lamellar structure. Separately, Tang et al. [40] utilized sodium molybdate as the molybdenum source, thioacetamide as the sulfur source, hydroxylamine hydrochloride as the reducing agent, and cetyltrimethylammonium bromide as the structure-directing agent. Through hydrothermal synthesis, they successfully obtained nanoflower-like MoS_2_ architectures approximately 1–2 μm in diameter.

Chemical vapor deposition (CVD) operates on the principle of initiating chemical reactions among gaseous precursors on engineered solid substrates to synthesize materials. This technique is widely employed for the scalable fabrication of thin films and layered materials. However, its utility is constrained by high deposition temperatures, which limit substrate compatibility, and the generation of toxic byproducts during the process [41,42]. Li et al. [43] used the CVD method to prepare both undoped and chlorine-doped MoS_2_ materials. After pre-treatment, place the substrate and sulfur powder in the high-temperature (850 °C) and low-temperature (200 °C) zones of a quartz tube. Use argon as the carrier and protective gas. Heat the high-temperature zone for 1 min, then cool naturally to complete the CVD growth (Figure 4b). Lee et al. [44] synthesized a large-area monolayer and few-layer MoS_2_ on SiO_2_/Si substrates using molybdenum trioxide (MoO_3_) and sulfur (S) powders as precursors. Incorporating nanomaterials such as reduced graphene oxide (rGO) during substrate pretreatment effectively promoted the layer-by-layer growth of MoS_2_. This approach enables controlled growth of two-dimensional materials on amorphous insulating substrates via nucleation engineering, offering a promising strategy for scalable production of TMDs.

Table 1 presents the advantages and disadvantages of common synthesis techniques for MoS_2_. Various methods for obtaining MoS_2_ particles with different morphologies and spatial structures each have their advantages. While mechanical exfoliation offers a higher yield, its slow rate makes it more suitable for fundamental research. Liquid-phase exfoliation and hydrothermal methods, known for their cost-effectiveness, are commonly used in electrode preparation. CVD is ideal for growing large-area MoS_2_ but involves high transfer costs to specific substrates. Given MoS_2_’s structural complexity, current synthetic efforts focus on developing novel MoS_2_ composite nanostructures to increase specific surface area, active sites, and ion transport efficiency.

## 3. Electrochemical Detection Based on MoS_2_ Electrode Materials

The electrochemical method primarily relies on the electrochemical properties of material components and their changing patterns for analysis and detection. The core principle lies in the ability of electrochemical sensors to directly detect changes in electrical signals, such as current, voltage, resistance, and potential. These changes essentially reflect the characteristics and behaviors of substances during electrochemical reactions. Specifically, electrochemical sensors are typically composed of a working electrode, a reference electrode, and a counter electrode. When the target substance comes into contact with the working electrode, a redox reaction occurs on the electrode surface. This reaction process is accompanied by charge transfer, leading to changes in the electrical signal. These changes in the electrical signal can be directly detected and analyzed by the detection instrument without the need for additional signal conversion steps [45,46,47,48]. Electrochemical techniques, based on the influence of HMIs on electrical signals, can primarily be categorized into potentiometry, amperometry, voltammetry, coulometry, impedance spectroscopy, and electrochemiluminescence techniques. These techniques each have distinct characteristics and are suited for different detection requirements [49].

### 3.1. Voltammetry

Voltammetry is the most widely used electrochemical method for determining and detecting HMIs in various complex environmental matrices. This technique applies different potential differences between the working electrode and the reference electrode, and measures the corresponding current to obtain the current-voltage curve. It possesses characteristics such as high accuracy, lower detection limits, and high sensitivity. Although voltammetry encompasses multiple technical types, its core operational principle is based on applying varying potentials and recording the responsive current [50,51]. The performance of electrochemical sensors is critically dependent on the working electrode material. MoS_2_ nanosheets’ exposed sulfur edges and defects offer ample active sites for heavy metal ions to adsorb and undergo electrochemical reduction, thereby amplifying the Faraday current response. Moreover, 1T-MoS_2_’s superior conductivity not only accelerates electron transfer but also diminishes charge-transfer impedance, expediting reaction kinetics. This is vital for producing clear, sharp stripping peaks in voltammetry and other key techniques.

**Figure 5:**
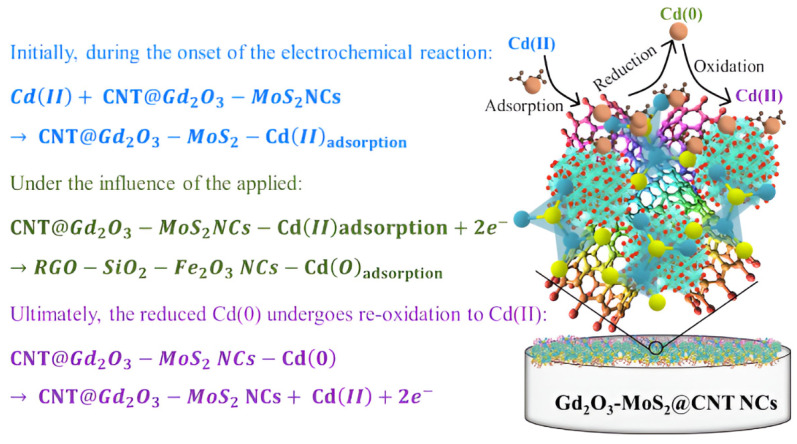
Cd(II) sensing based on Gd_2_O_3_-MoS_2_@CNT NCs. Reproduced with permission from ref. [53], Copyright 2024, Elsevier.

Cyclic voltammetry (CV): This method applies a triangular wave potential signal between the working electrode and reference electrode, while simultaneously recording the relationship curve between the obtained current and applied potential. Key parameters such as reduction peak potential, oxidation peak potential, and corresponding current values can be extracted from this curve. CV holds significant value for optimizing analytical parameters and preliminary identification of analytes, but its sensitivity is limited for trace-level detection of HMIs [52]. Mashkoor et al. [53] successfully prepared carbon nanotube-modified Gd_2_O_3_-MoS_2_ ternary nanocomposites (Gd_2_O_3_-MoS_2_@CNT NCs) via hydrothermal synthesis. They achieved highly sensitive electrochemical detection of Cd^2+^ in water using CV, while adsorption experiments demonstrated synergistic purification capabilities. As shown in Figure 5. At pH 6, this material achieved 90% adsorption efficiency for 100 mg/L Cd^2+^ with a maximum adsorption capacity of 187.10 mg/g. The modified electrode demonstrated a linear response within 5–110 ppb, exhibiting a sensitivity of 5.396 × 10^−2^ μA/ppb cm^2^ and a detection limit of 2.363 ppb. It also displayed strong anti-interference capability. This study provides new insights for developing high-efficiency materials to detect and recover heavy metals.

Pulse Voltammetry: This technique is used to study reaction kinetics and electrochemical processes in solutions. Its principle involves applying a series of pulsed voltages or currents with varying shapes and amplitudes to trigger electrochemical reactions in the solution, followed by measuring the resulting current response at the electrode. Based on pulse methodologies, this technique can be further categorized into normal pulse voltammetry, reverse pulse voltammetry, and differential pulse voltammetry (DPV). DPV is widely used for heavy metal ion detection due to its exceptional sensitivity. This method applies staircase-shaped pulse voltage signals instead of traditional linear scans, significantly enhancing the signal-to-noise ratio (SNR) of current responses. Its unique differential current measurement mechanism not only effectively distinguishes redox characteristic peaks of targets but also enables precise quantification of trace metal ions [54]. Aswathi et al. [55] prepared MoS_2_-modified glassy carbon electrodes via the solvent exfoliation method and achieved ultra-sensitive selective detection of Hg^2+^ using DPV. The results demonstrated that the MoS_2_/GCE exhibited a detection limit as low as 0.000001 nM for Hg^2+^ in 1M HCl electrolyte, showing excellent selectivity for Hg^2+^ with minimal current response to most other metal ions except Ag^+^. This study provides a reliable solution for the rapid and portable detection of HMIs in complex environmental samples, such as high-salinity seawater.

Stripping Voltammetry: This combined technique detects analytes through a two-step process: first, electrodepositing target species from solution onto the working electrode surface, then applying voltammetric potentials to strip the deposited material back into solution for analysis. Based on the stripping mechanism, it is categorized into anodic stripping voltammetry (ASV) and cathodic stripping voltammetry (CSV). ASV uses anodic potential scanning to oxidize and release analytes, while CSV employs cathodic potential scanning to reduce and release them [56]. The integration of stripping techniques with voltammetry has led to innovative detection methods, primarily including three representative modes: Linear Sweep Anodic Stripping Voltammetry (LSASV), Differential Pulse Anodic Stripping Voltammetry (DPASV), and Square Wave Anodic Stripping Voltammetry (SWASV). By combining the preconcentration effect of the stripping process with the high sensitivity of pulse measurements, these techniques demonstrate significant advantages for efficient trace-level heavy metal ion detection [57]. Zhang et al. [58] developed a glassy carbon electrode modified with Mn-MoS_2_/MWCNTs/Nafion and integrated it with an electrochemical flow analysis system for Pb^2+^ detection in water. Using DPASV, the system achieved a detection limit of 0.08 μg/L with a linear range of 0.2–100 μg/L. Recovery rates for real water samples reached 97.3–105.6%. The platform demonstrated excellent accuracy and anti-interference capability in practical water testing, proving suitable for on-site rapid detection in environmental water bodies with substantial potential for continuous monitoring of heavy metals in natural waters. Xu et al. [59] developed a high-sensitivity electrochemical method for Hg^2+^ detection by constructing an atomic-level MoS_2_/NiS_2_ heterojunction. The MoS_2_/NiS_2_ composite was synthesized hydrothermally, and SWASV under optimized conditions achieved a detection limit of 0.0111 μM with a sensitivity of 459.13 μA μM^−1^ cm^−2^. This study highlights the critical role of atomic-level interface engineering in enhancing electroanalytical performance, providing important references for environmental monitoring and precision analysis technologies.

### 3.2. Impedance Measurement Techniques

Impedance measurement techniques analyze electroactive species in solutions by measuring their impedance characteristics. Among the most widely employed methods for determining analyte concentrations in aqueous solutions are electrochemical impedance spectroscopy and alternating current voltammetry. Electrochemical impedance spectroscopy applies a small-amplitude sinusoidal voltage or current to an electrochemical cell and measures the corresponding current or voltage response to obtain impedance spectra. This technique provides detailed information about the electrode–electrolyte interface, including double-layer capacitance, charge transfer resistance, and diffusion impedance [60]. Widely applied in biological and chemical fields, impedance measurement techniques offer a cost-effective and simple approach for sensitive detection of toxic metal ions in biological and chemical matrices compared to other electrochemical methods [61]. Neethipathi et al. [62] developed a flexible electrochemical sensor based on MoS_2_-modified screen-printed carbon electrode (SPCE) for Cu^2+^ detection in water. MoS_2_ nanomaterials synthesized hydrothermally were drop-cast onto the working electrode area to fabricate the sensor. CV and DPV were employed for quantitative analysis of Cu^2+^ concentration, while electrochemical impedance spectroscopy compared the performance of MoS_2_-modified SPCE with a conventional glassy carbon electrode. The sensor achieved a detection limit of 5.43 μM for Cu^2+^ with a broad linear range of 5 μM–5 mM. Compared with a conventional glassy carbon electrode, the SPCE-based sensor demonstrates superior sensitivity and linear response, proving suitable for copper ion detection in practical water samples. This advancement provides a crucial technical pathway for the practical implementation of flexible electrochemical sensors in environmental monitoring. Although there are currently no specific examples of using MoS_2_ for impedance detection of heavy metal ions, we believe that further research and exploration in this field will yield fruitful results.

### 3.3. Potentiometry

Potentiometric analysis measures the potential difference between an indicator electrode and a reference electrode under near-zero current conditions. This potential difference exhibits linear proportionality to the concentration of HMIs, enabling quantitative analysis of heavy metals [63]. Unlike other electrochemical techniques, potentiometry measures ion activity rather than concentration in solutions for qualitative and quantitative analysis. Characterized by low cost, rapid response, non-destructive operation, simplicity, applicability to precious samples, and ease of automation, it has been extensively applied for heavy metal ion detection in complex environmental matrices [64]. Huang et al. [65] developed a high-performance potentiometric sensor for Pb^2+^ using polysulfoaminoanthraquinone (PSA) as a solid-state ionophore. For the first time, conductive PSA polymer nanoparticles served as the solid-state ionophore, where amino groups, carbonyl groups, and sulfonate groups in their molecular structure synergistically achieved Pb^2+^-specific recognition by forming a rigid coordination cavity. The sensor exhibited a detection limit of 1.6 × 10^−7^ M with a linear range of 10^−1.63^–10^−1.6^ M, a 16-s response time, and a 5-month operational lifespan. By optimizing the solid-state ionophore design and component synergy, this sensor overcomes limitations of traditional Pb^2+^ sensors, such as poor stability and weak anti-interference capability.

### 3.4. Electrochemiluminescence Techniques

Electrochemiluminescence (ECL) is an analytical technique based on light emission triggered by electrochemical reactions. This method generates free radicals or other reactive species through electrolysis, which subsequently undergo chemical reactions accompanied by photon emission. The core principle lies in initiating luminescence via electrochemical processes to detect target analytes [66,67]. ECL reactions involve two key steps: electrochemical reaction and chemiluminescence. During the electrochemical step, redox processes at the electrode surface generate reactive intermediates. In the subsequent chemiluminescent step, these intermediates react with other components in the system to form excited-state species. When these excited-state species return to their ground state through de-excitation, light at specific wavelengths is emitted. This process not only efficiently converts chemical energy into light but also offers high sensitivity, low background signal, and tunable electrochemical potential, enabling widespread applications in biomedical analysis, food testing, and environmental monitoring [68,69]. Cui et al. [70] constructed a label-free bifunctional ECL sensor based on Ru(bpy)_3_^2+^-functionalized metal–organic frameworks (Ru-MOFs) and strand displacement amplification (SDA) for ultrasensitive simultaneous detection of Hg^2+^ and Ag^+^ in water. The sensor achieved detection limits of 0.00032 pM for Hg^2+^ and 0.00298 pM for Ag^+^, with sensitivity enhanced by 2–3 orders of magnitude compared to existing technologies. High selectivity was maintained in seawater containing nine interfering ions. Spike recovery rates in real seawater samples ranged from 93.43% to 105.49%, establishing an accurate and efficient analytical platform for marine heavy metal pollution monitoring. In this work, we have cited examples of other materials to provide reference and inspiration for the development of MoS_2_-based electrochemical sensors. These examples highlight the application strategies and performance advantages of different materials in techniques such as potentiometry, electrochemiluminescence, and impedimetric detection. They offer valuable insights and directions for exploring and refining the design of MoS_2_-based sensors. In this work, we have cited examples of other materials to provide reference and inspiration for the development of MoS_2_-based electrochemical sensors. These examples highlight the application strategies and performance advantages of different materials in techniques such as potentiometry, electrochemiluminescence, and impedimetric detection. They offer valuable insights and directions for exploring and refining the design of MoS_2_-based electrochemical sensors.

## 4. Electrochemical Sensing Applications of MoS_2_-Based Composites

**Figure 6:**
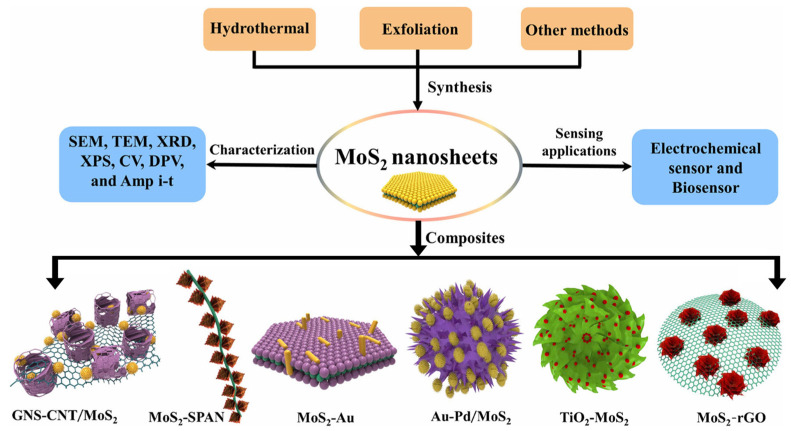
Schematic representations for the preparation of MoS_2_-based nanocomposites. Reproduced with permission from ref. [75], Copyright 2019, Springer Nature.

Although MoS_2_-based electrochemical sensors offer certain advantages, they still face limitations in selectivity and sensitivity. To meet the demand for more sensitive, rapid, and stable heavy metal detection, researchers have proposed various composite strategies. MoS_2_ is combined with other conductive nanomaterials and functional building blocks, such as metallic nanoparticles, metal oxides, and organic compounds, to form new nano-composites. This design not only enhances the material’s conductivity but also leverages the synergistic effects of its components, overcoming individual material defects. It creates electrochemical sensing materials with superior overall performance, offering an effective solution for high-sensitivity detection of heavy metal ions and other targets [71,72,73,74]. As shown in Figure 6. This section introduces the common types of MoS_2_ composites and their applications in electrochemical sensing (Table 2).

### 4.1. MoS_2_/Metal Nanoparticle Composites

Metal nanoparticles (MNPs), particularly noble metal systems such as platinum (Pt), gold (Au), silver (Ag), and palladium (Pd), demonstrate significant advantages in electrochemical sensing. Their high specific surface area effectively increases the density of active sites on electrodes, thereby enhancing detection sensitivity. Concurrently, nanoscale effects impart superior mass transfer kinetics and electron transfer rates, substantially optimizing electrode response performance. In functional design, MNPs readily undergo surface modification to construct specific recognition interfaces. Through catalytic enhancement of redox reactions and reinforcement of electron transport pathways, they synergistically improve sensing efficiency and selectivity [76,77,78]. Leveraging these properties, MNPs serve as key components in composites with two-dimensional materials, enabling high-precision quantitative detection of trace HMIs through electrochemical technologies. El-Raheem et al. [79] developed a carbon paste electrode modified with polyurethane (PU)-incorporated platinum nanoparticles for sensitive and selective voltammetric determination of free copper ions in biological samples. Experimental optimization of electrode composition, supporting electrolyte, and scan rate revealed a linear response within 100–1000 ng/mL Cu^2+^ concentration, achieving a detection limit of 16.72 ng/mL. This novel electrode provides a sensitive, selective, and cost-effective option for copper ion detection. Zhang et al. [80] developed a portable analytical system based on gold nanoparticle (AuNP)-alloy modified screen-printed carbon electrodes (AuASEs) for on-site detection of Cd^2+^ in environmental water samples. This system offers low cost, portability, and operational simplicity, making it suitable for rapid monitoring during environmental emergencies. In 0.1 M phosphate buffer (pH 5.5), the AuASEs achieved a detection limit of 2.6 ppb for Cd^2+^, with linear current response to Cd^2+^ concentration from 8.4 ppb to 500 ppm, demonstrating excellent sensitivity, selectivity, and reproducibility.

**Figure 7:**
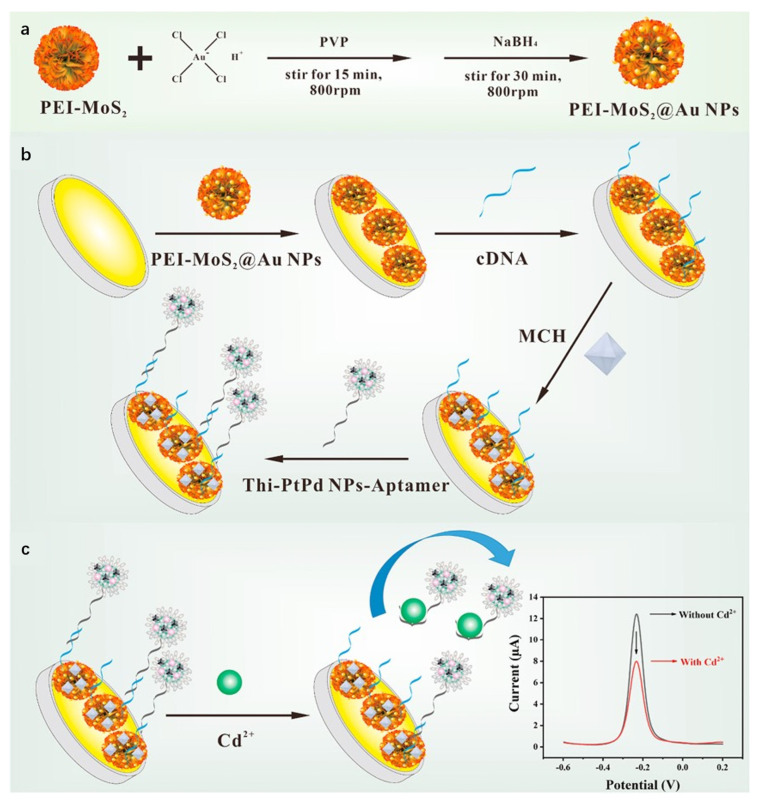
(**a**) Preparation of PEI-MoS_2_@Au NPs; (**b**) Preparation of aptasensor; (**c**) Detection principle of the electrochemical aptasensor for Cd^2+^. Reproduced with permission from ref. [82], Copyright 2022, Elsevier.

Studies demonstrate that composite materials formed by integrating MoS_2_ with metal nanoparticles retain the intrinsic properties of both components while significantly enhancing overall performance through synergistic effects. Liu et al. [81] developed an electrochemical sensor based on MoS_2_/Au heterojunction nanostructures for highly sensitive detection of trace Hg^2+^ in wastewater. Gold nanoparticles were deposited onto MoS_2_ nanosheets via a self-assembly method to form MoS_2_/Au heterojunction composites. The heterostructure generated mid-gap states through hybridization of Au 5d and Mo 3d orbitals, enhancing electron conductivity and electrocatalytic activity. Using DPV, the sensor achieved a linear range of 0.0004 ppb to 0.5 ppm with a detection limit of 0.0004 ppb, and was successfully applied to real wastewater samples. This study demonstrates that MoS_2_/Au composites effectively leverage the catalytic activity of metal nanoparticles and interfacial advantages of 2D materials, showing significant potential for electrochemical sensing applications. Li et al. [82] constructed an electrochemical aptasensor using PEI-MoS_2_@Au NPs and Thi-PtPd NPs core–shell spheres for ultrasensitive Cd^2+^ detection. As shown in Figure 7. Molybdenum disulfide nanoflowers served as substrates for in situ growth and loading of gold nanoparticles, functionalized with polyethylenimine to form three-dimensional flower-like nanocomposites. The PEI-MoS_2_@Au NPs-modified gold electrode functioned as the sensing interface, immobilizing cDNA via Au-S bonds. Signal probes were fabricated by loading Cd^2+^-specific aptamers and electroactive thionine onto PtPd NPs core–shell structures (Thi-PtPd NPs-aptamer). DPV quantified current variations, exhibiting linearity from 1 × 10^−3^ nM to 1 × 10^2^ nM with a detection limit of 2.34 × 10^−4^ nM, achieving ultra-sensitive Cd^2+^ analysis. With significant potential for food testing and environmental monitoring applications, it provides a novel approach for highly sensitive heavy metal analysis.

### 4.2. MoS_2_/Conductive Polymer Composites

Conductive polymers (CPs), as a class of critical organic functional materials, exhibit metal-like electrochemical properties coupled with multiple advantages. These include straightforward synthesis, diverse chemical structures, exceptional biocompatibility, and tunable conductivity [83]. The breakthrough discovery in conductivity originated with polyacetylene (PA), whose electrical conductivity increased by 10 orders of magnitude upon iodine vapor oxidation doping, transitioning it from an insulator to a conductor. Subsequently, numerous high-performance conductive polymers were developed, including polyaniline (PANI), polypyrrole (PPy), polythiophene (PT), and poly(aminonaphthalene). Through tailored synthesis strategies, these materials form diverse nanostructures, such as nanospheres, nanowires, and nanorods, further expanding their application potential in energy storage, sensors, and biomedicine [84,85]. Electrochemical sensors based on conductive polymers are currently attracting significant research interest and finding widespread application. Studies demonstrate that these conductive polymers offer distinct advantages, including high selectivity for target molecules, enabling faster detection times. Zhang et al. [86] developed a screen-printed carbon electrode sensor utilizing a phytic acid-functionalized polypyrrole (PA-PPy) composite. This sensor achieved highly sensitive detection of Pb^2+^ via in situ polymerization. Under optimized conditions using DPASV, the sensor exhibited a linear detection range for Pb^2+^ of 10–600 nM, with a detection limit as low as 0.43 nM. This limit is significantly lower than the threshold defined by the World Health Organization (WHO). Setiyanto et al. [87] developed a highly selective electrochemical sensor based on ion-imprinted polyaniline (IIPANI) for the detection of Ni^2+^ in water. The sensor utilized aniline as the functional monomer, electrochemically deposited onto a bismuth-modified carbon paste electrode (CPE-Bi), endowing it with high affinity and selectivity for Ni^2+^. Experimental parameters, including the number of electropolymerization cycles, aniline concentration, and solution pH, were optimized. Results demonstrated that the sensor exhibited a linear response to Ni^2+^ concentrations ranging from 0.01 to 1.00 mM, with a limit of detection of 0.00482 mM. Furthermore, the sensor accurately detected Ni^2+^ even in the presence of high concentrations of interfering ions and demonstrated good reproducibility and repeatability under the optimized conditions.

**Figure 8:**
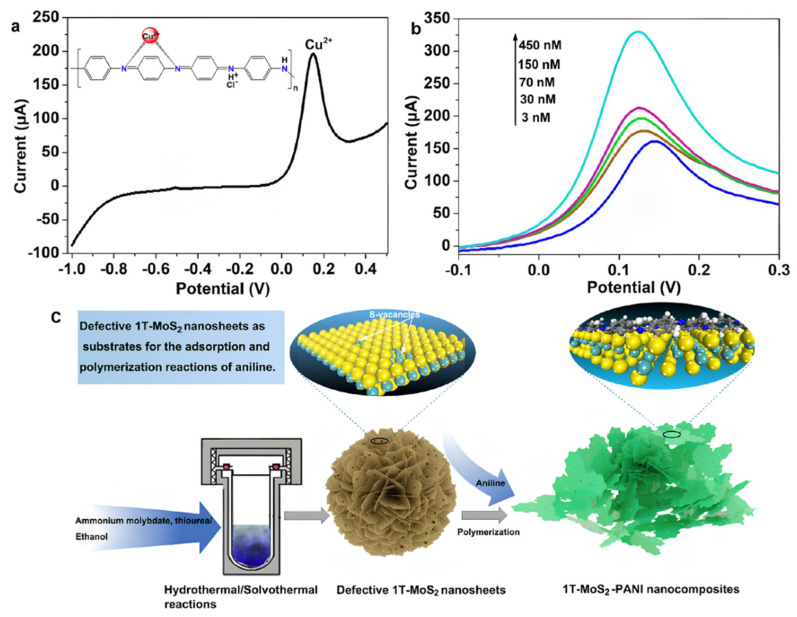
(**a**) Detection plots of 0.15 μM Ni^2+^, Cu^2+^, Pb^2+^, Cd^2+^, and Hg^2+^ using 1T-MoS_2_-PANI/GCE. The inset shows the possible interaction architecture between Cu^2+^ and PANI. (**b**) Detection plots of the 1T-MoS_2_-PANI/GCE for different concentrations of Cu^2+^. (**c**) Fabrication of 3D Porous 1T-MoS_2_−PANI composites. Reproduced with permission from ref. [88], Copyright 2023, American Chemical Society.

Through compositing with conductive polymers, MoS_2_ not only significantly enhances the material’s conductivity and catalytic activity but also further improves the sensor’s selectivity and sensitivity. Gan et al. [88] prepared metallic-phase 1T-MoS_2_ and formed a three-dimensional porous PANI/MoS_2_ composite via acid-doped in situ polymerization for the electrochemical detection of Cu^2+^. As shown in Figure 8. Combined experimental and density functional theory (DFT) calculations confirmed that PANI preferentially anchors at sulfur vacancy clusters via Mo–N covalent bonding and embeds within the van der Waals gaps of MoS_2_ layers in a planar configuration, significantly enhancing material stability. Using linear sweep voltammetry (LSV) for detection, the study demonstrated that this composite-modified glassy carbon electrode (1T-MoS_2_/PANI/GCE) exhibited a more than twofold increase in response current towards Cu^2+^ compared to single-component electrodes (1T-MoS_2_/GCE or PANI/GCE). The sensor achieved an ultralow detection limit of 0.33 nM within a broad linear range of 3–450 nM, highlighting the synergistic optimization of electron transport and interfacial adsorption kinetics. This synergy arises from the core advantages of PANI’s coordination recognition capability coupled with the conductive network of 1T-MoS_2_.

### 4.3. MoS_2_/Carbon-Based Material Composites

Carbon-based materials constitute a versatile class of materials formed by carbon atoms arranged in diverse configurations, exhibiting a wide range of morphologies and physicochemical properties. Representative examples include carbon nanoparticles (CNPs), carbon nanotubes (CNTs), carbon nanofibers (CNFs), carbon quantum dots (CQDs), graphene, and its derivatives [89]. These materials offer significant advantages in constructing electrochemical sensors due to their low cost, high chemical stability, excellent mechanical strength, remarkable thermal stability, and outstanding electrical conductivity. Their unique electron transport properties and tunable surface chemistry provide ideal platforms for enhancing sensor sensitivity, selectivity, and response speed [90,91]. Simpson et al. [92] described the preparation and characterization of CNPs and their application in the electrochemical detection of HMIs in aqueous solutions. The study optimized the modification conditions of CNPs on glassy carbon electrodes and the electrochemical detection parameters. Using SWASV for the detection of Pb^2+^ and Cu^2+^, limits of detection of 0.3 ppm and 0.5 ppm, respectively, were achieved. This work demonstrates the potential of CNPs for electrochemical heavy metal ion detection in water for the first time. Li et al. [93] reported a modified multi-walled carbon nanotube (MWCNT) material functionalized through nitrogen doping and thiol-group modification for the simultaneous detection of Cd^2+^ and Pb^2+^ ions in aqueous solutions. The modified MWCNTs, prepared via chemical oxidation, hydrothermal treatment, and diazotization reactions, exhibited excellent electrochemical properties. This material demonstrated high sensitivity and selectivity using SWASV, achieving detection limits of 0.4 μg/L for Cd^2+^ and 0.3 μg/L for Pb^2+^ within a linear range of 2–50 μg/L. The modified MWCNTs also showed satisfactory stability and repeatability under continuous operation.

**Figure 9:**
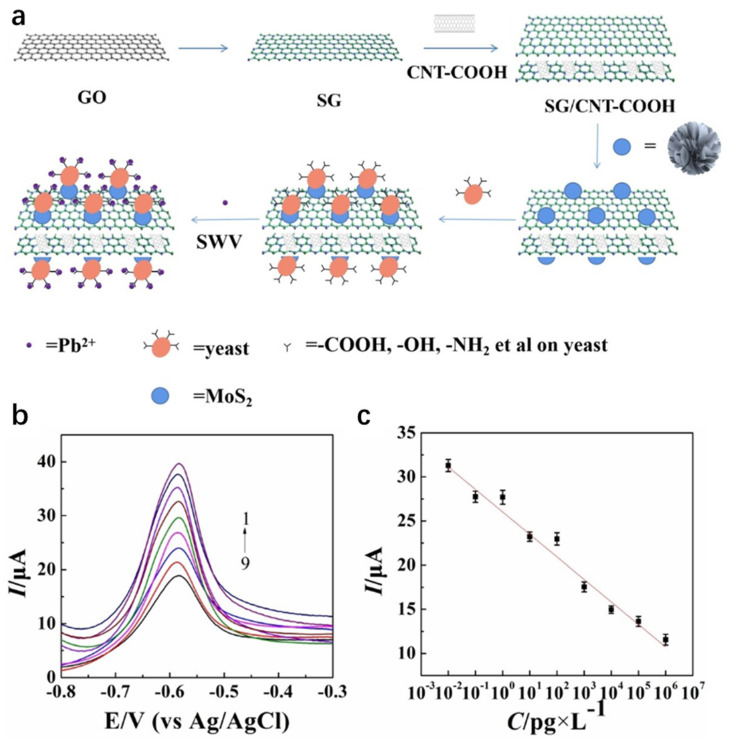
(**a**) The formation of SG/CNTCOOH/MoS_2_/yeast and the application of as-prepared SG/CNTCOOH/MoS_2_/yeast in the detection of Pb^2+^. (**b**) SWV response currents of SG/CNT COOH/MoS_2_/yeast/GCE sensor in 0.1 M ABS (pH = 5.5) with a deposition time of 180 s and a deposition potential of 1.2 V containing different Pb^2+^ concentrations (1–9): 0.01, 0.1, 1, 10, 102, 103, 104, 105 and 106 pg/L. (**c**) Standard curves of Pb^2+^ with different concentrations. Error bars: n = 3. Reproduced with permission from ref. [94], Copyright 2023, Wiley.

The synergistic integration of carbon-based materials and MoS_2_ capitalizes on the former’s high conductivity and stability, combined with the latter’s superior electrochemical activity and catalytic properties. In Figure 9. Yin et al. [94] engineered a sulfur-doped graphene (SG)/carboxylated carbon nanotube (CNT-COOH)/MoS_2_/yeast composite-modified glassy carbon electrode (GCE) via integrated hydrothermal-sonication synthesis for ultrasensitive electrochemical Pb^2+^ detection. The dual-carbon scaffold (SG/CNT-COOH) establishes a conductive network with enriched active sites, whereas MoS_2_ enhances electrocatalytic activity. Concurrently, hydroxyl/amino/thiol groups (-OH/-NH_2_/-SH) on yeast enable Pb^2+^-specific capture through ion-exchange and surface complexation mechanisms. The composite material exhibits high responsiveness in detecting low-concentration lead ions, with a detection limit as low as 2.61 × 10^−15^ g/L and a linear range of 10^−6^ to 10^−14^ g/L. It also shows potential for practical applications in real serum samples. This work breaks through the detection limit via a biomass-nanocarbon-chalcogenide ternary synergistic strategy, offering a new approach for heavy metal monitoring in complex matrices. Kancharla et al. [95] synthesized MoS_2_ quantum dots (MSQDs) via mechanical shear exfoliation and constructed a low-cost, disposable electrochemical sensor based on a pencil graphite electrode (PGE) (MSQD/PGE) for the highly sensitive detection of Pb^2+^. Detection of Pb^2+^ employed ASDPV, with experimental parameters including pH, deposition potential, and deposition time optimized. The sensor exhibited a linear response to Pb^2+^ over the concentration range of 5.66 to 491.93 nM, achieving a detection limit of 1.96 nM. It also demonstrated high selectivity in the presence of other metal ions. Analysis of real samples (agricultural soil and groundwater) yielded an average Pb^2+^ recovery rate of approximately 99.50%, confirming its practical utility. This study provides a novel approach for the on-site, rapid detection of trace Pb^2+^ in environmental samples and offers a cost-effective alternative for field monitoring of heavy metal pollution. Rana et al. [96] synthesized a MoS_2_-rGO 2D layered nanocomposite via hydrothermal synthesis and constructed a low-cost electrochemical sensor (MoS_2_-rGO/CPE) based on a carbon paste electrode for highly sensitive Hg^2+^ detection. CV and DPASV were used to detect Hg^2+^, with experimental parameters like pH, deposition potential, and time optimized. Compared to a bare electrode, the MoS_2_-rGO modified electrode shows a 103% higher oxidation peak current response. The sensor has a good linear response to Hg^2+^ at 1.0–10.0 µM, a detection limit of 1.6 µM, and high selectivity in the presence of other metal ions. The enhanced sensing performance of the MoS_2_-rGO modified sensor is attributed to the synergistic effect of MoS_2_ and rGO. This work demonstrates the immense potential of this sensor in the development of the next generation of heavy metal ion sensors. Although CPE have the advantages of easy modification and low cost, they also have several noteworthy drawbacks. A primary concern is their mechanical stability; the soft, paste-like matrix is more prone to physical erosion in stirred solutions or gradual deformation, which can lead to unstable currents and complicate long-term measurements. Furthermore, achieving highly reproducible surfaces can be challenging. The surface reproducibility is highly dependent on the operator’s skill in packing and renewing the paste, where inconsistent packing density or surface smoothing can result in variable electroactive area and background noise, making inter-electrode comparison less reliable than with polished solid electrodes like glassy carbon.

### 4.4. MoS_2_/Metal Oxide Composites

Metal oxides, binary compounds composed of metal and oxygen elements, offer versatile synthesis routes, including solution-based processes, vapor deposition, and controlled oxidation of metals, enabling precise tuning of morphology and structure for specific applications. Owing to their high specific surface area, enhanced surface reactivity, pronounced interfacial effects, and favorable biocompatibility, metal oxide nanomaterials have found extensive application in electrochemical sensing [97]. By modulating electronic structures and leveraging multivalent redox activity, coupled with diverse nanostructural designs, these materials significantly enhance the preconcentration capacity and catalytic conversion efficiency for trace HMIs, thereby playing a critical role in electrochemical sensing platforms [98,99]. Among the most common metal oxides employed for heavy metal detection are iron oxides with tailored morphologies. Lee et al. [100] pioneered the application of Fe_2_O_3_ nanoparticles in electrochemical sensing. They constructed a sensor using a glassy carbon electrode modified with an iron oxide/graphene (Fe_2_O_3_/G) nanocomposite—synthesized via a solvothermal method—combined with in situ plated bismuth film (Bi) for the simultaneous detection of trace Zn^2+^, Cd^2+^, and Pb^2+^. Employing DPASV, the modified electrode demonstrated enhanced electrochemical catalytic activity and high sensitivity toward these HMIs, attributed to the synergistic effect between graphene and Fe_2_O_3_ nanoparticles. Under optimized conditions, the electrode exhibited linear responses for all three metal ions over a concentration range of 1–100 μg/L, achieving detection limits of 0.11 μg/L for Zn^2+^, 0.08 μg/L for Cd^2+^, and 0.07 μg/L for Pb^2+^. Zhang et al. [101] developed an electrochemical sensor using alkaline-functionalized Fe_2_O_3_@SiO_2_ for simultaneous detection of Cd^2+^, Pb^2+^, Cu^2+^, and Hg^2+^ in milk. The alkali treatment confers permanent negative surface charges on SiO_2_, enabling electrostatic preconcentration of heavy metal cations. Under optimized conditions, linear detection ranges were 0.1–100 μM (Cd^2+^), 0.1–80 μM (Pb^2+^), 0.1–80 μM (Cu^2+^), and 0.1–100 μM (Hg^2+^), with individual detection limits of 16.5–79.4 nM. This integrated approach combines magnetic extraction with direct electrochemical detection, eliminating separate pretreatment steps. Recoveries in spiked milk samples ranged from 96.0% to 104.3%.

**Figure 10:**
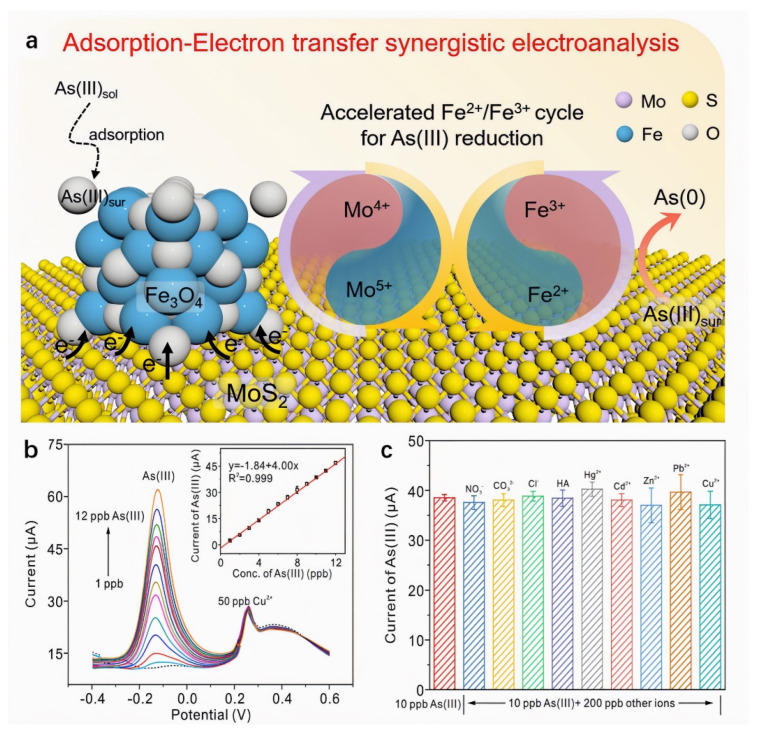
(**a**) Detection strategy of As(III) on Fe_3_O_4_/MoS_2_ nanocomposite based on the synergistic effect of the adsorption of Fe_3_O_4_ and the enhanced electron transfer induced by the accelerated Fe^2+^/Fe^3+^ cycle. (**b**) The SWASV response of Fe_3_O_4_/MoS_2_ to As(III) at a concentration of 1–10 ppb in the presence of 50 ppb Cu^2+^. The inset in (**b**) shows the corresponding linear relationship between current and As(III) concentration. (**c**) The current of 10 ppb As(III) at Fe_3_O_4_/MoS_2_ in the presence of 50 ppb anions, cations, and humic acid, respectively. Reproduced with permission from ref. [102], Copyright 2022, Elsevier.

Studies demonstrate that compositing MoS_2_ with metal oxides enhances electron transfer efficiency through synergistic effects, improving both conductivity and material stability. Xia et al. [102] developed an electrochemical sensor based on Fe_3_O_4_/MoS_2_ nanocomposites for highly sensitive As(III) detection. Combined XPS valence analysis and DFT calculations revealed a synergistic mechanism where Mo^4+^ sites on MoS_2_ accelerate the Fe^2+^/Fe^3+^ redox cycling in Fe_3_O_4_ via electron transfer. A built-in electric field at the heterointerface further facilitates electron migration, overcoming the limitations of Fe_3_O_4_’s poor conductivity and sluggish As(III) reduction kinetics. Using SWASV under optimized conditions, the sensor achieved a high sensitivity of 4.16 μA/ppb and a low detection limit of 0.021 ppb, outperforming most noble-metal-based sensors. As shown in Figure 10. It also exhibited excellent stability, anti-interference capability, and applicability in real water matrices, providing a new platform for electrochemical As(III) monitoring. Gao et al. [103] fabricated a glassy carbon electrode sensor modified with nano-Fe_3_O_4_/MoS_2_ nanocomposite and Nafion binder via drop-casting for Cd^2+^ detection in seawater. The sensor leverages synergistic contributions: Fe_3_O_4_ nanoparticles provide strong adsorption capacity and electrical conductivity, MoS_2_ nanosheets enhance structural stability and prevent agglomeration, while the Nafion matrix enables cation exchange and confers anti-fouling properties. Precise quantification was achieved using DPV. Under optimized conditions, the sensor exhibited a linear response from 5 to 300 μg/L with a detection limit of 0.053 μg/L. Recovery rates of 99.2–102.9% in spiked seawater samples demonstrated high accuracy and robustness against matrix effects. This work provides a practical and cost-effective solution for monitoring cadmium pollution in marine environments.

### 4.5. MoS_2_/Chitosan Composites

Chitosan is a deacetylated derivative of chitin, featuring abundant free hydroxyl (-OH) and amino (-NH_2_) groups along its polymer chains. This molecular structure confers unique biocompatibility, biodegradability, and environmental benignity. The functional groups further enable exceptional adsorption capacity, film-forming ability, moisture retention, and polyelectrolyte behavior. Moreover, chitosan’s intrinsic nitrogen richness and renewable sourcing establish it as an ideal precursor for fabricating N-doped carbon electrodes. Its three-dimensional porous network and high specific surface area facilitate effective functionalization [104,105]. Singh et al. [106] developed a portable potentiometric device based on a chitosan-graft-polyaniline (Chit-g-PANI) composite electrode for highly selective Cu^2+^ detection. The hybrid material was synthesized through chemical oxidative grafting of polyaniline onto the chitosan backbone, creating accessible -NHCOCH_3_ binding sites. The electrode demonstrated a linear detection range from 1 to 1000 ppm for Cu^2+^ with a detection limit of 13.77 ppm, exhibiting excellent sensitivity and selectivity in environmental matrices.

**Figure 11:**
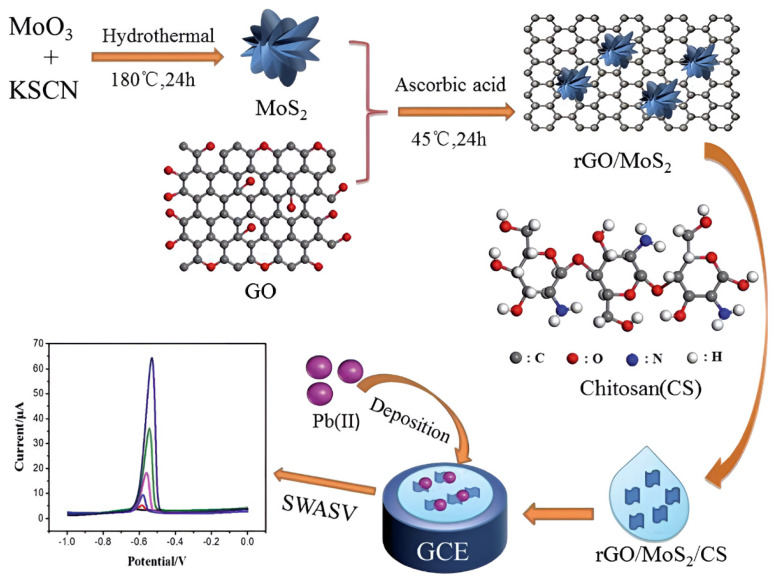
The constructed sensor based on GCE modified with rGO/MoS_2_/CS and electrochemical analysis process for Pb^2+^. Reproduced with permission from ref. [107], Copyright 2021, Royal Society of Chemistry.

Notably, MoS_2_ has emerged as a critical component for enhancing chitosan-based electrochemical sensors due to its tunable bandgap, high specific surface area, and abundant edge-active sites. Constructing heterostructures with chitosan enables synergistic enhancement of both heavy metal ion preconcentration capacity and detection sensitivity. Guo et al. [107] developed an electrochemical sensor for ultrasensitive Pb^2+^ detection in tobacco leaves using a glassy carbon electrode modified with rGO, nanoflower-like MoS_2_, and chitosan (rGO/MoS_2_/CS/GCE). The fabrication protocol initiated with hydrothermal synthesis of MoS_2_ nanoflowers, followed by L-ascorbic acid reduction in graphene oxide and its subsequent compositing with MoS_2_. This composite was then dispersed in chitosan solution to form a homogeneous modifier suspension prior to drop-casting onto the GCE surface. As shown in Figure 11. Under optimized SWASV conditions, the sensor exhibited a linear response spanning 0.005 to 2.0 mM with a detection limit of 1.6 μM. The operational mechanism leverages trifunctional synergy: chitosan’s amino/hydroxyl groups enhance Pb^2+^ preconcentration through coordination, rGO facilitates rapid electron transfer via its conductive network, while the nanoflower MoS_2_ architecture provides abundant electroactive sites. This synergistic integration confers exceptional stability and anti-interference capability, establishing an effective analytical strategy for heavy metal detection in complex low-abundance matrices. Wang et al. [108] developed an electrochemical sensor for ultrasensitive mercury ion detection in aquatic systems using a glassy carbon electrode modified with chitosan/graphene oxide/MoS_2_/gold nanoparticles (CS/GO/MoS_2_/AuNPs). Electrochemical impedance spectroscopy and DPSV revealed a linear response range of 0.01–4 μg/L, with a detection limit of 5.8 ng/L. This sensor demonstrates exceptional sensitivity, low cost, and operational simplicity, offering a promising approach for monitoring trace mercury contamination in water.

**Table 2:** Various electrode materials for HMI detection.

Materials	Technique	Real Sample	Metal Ion	LOD	Detection Range	Ref.
Gd_2_O_3_-MoS_2_@CNT NCs	CV	Tap water	Cd^2+^	2.363 ppb	5–110 ppb	[53]
MoS_2_/GCE	DPV	Tap water and sea water	Hg^2+^	0.2 ppq	0.1 nM–0.2 mM	[55]
Mn-MoS_2_/MWCNTs/NA/GCE	DPASV	Tap water and lake water	Pb^2+^	0.08 μg/L	0.2–100 μg/L	[58]
MoS_2_/NiS_2_	SWASV	Not specified	Hg^2+^	0.0111 μM	0–4 μM	[59]
MoS_2_/SPCE	CV, DPV	Not specified	Cu^2+^	5.43 μM	5 μM–5 mM	[62]
MoS_2_/Au	DPV	Factory and environmental lab wastewater	Hg^2+^	0.0004 ppb	0.0004 ppb–0.5 ppm	[81]
PEI-MoS_2_@Au NPs	DPV	Tap water	Cd^2+^	2.34 × 10^−4^ nM	1 × 10^−3^ nM–1 × 10^2^ nM	[82]
1T-MoS_2_/PANI/GCE	LSV	Tap water	Cu^2+^	0.33 nM	3–450 nM	[88]
SG/CNT-COOH/MoS_2_/Yeast/GCE	SWV	Human serum	Pb^2+^	2.61 × 10^−15^ g/L	10^−6^–10^−14^ g/L	[94]
MSQD/PGE	ASDPV	Agriculture soil and ground water	Pb^2+^	1.96 × 10^−9^ M	5.66 × 10^−9^ M–491.93 × 10^−9^ M	[95]
MoS_2_-rGO/CPE	DPASV	Tap water	Hg^2+^	1.6 µM	1.0–10.0 µM	[96]
Fe_3_O_4_/MoS_2_	SWASV	Nanhu Lake and tap water	As(III)	0.021 ppb	1–10 ppb	[102]
Fe_3_O_4_/MoS_2_/Nafion/GCE	DPV	Seawater	Cd^2+^	0.053 μg/L	5–300 μg/L	[103]
rGO/MoS_2_/CS/GCE	SWASV	Tobacco leaves	Pb^2+^	0.0016 mM	0.005–2.0 mM	[107]
CS/GO/MoS_2_/AuNPs	DPSV	Not specified	Hg^2+^	5.8 ng/L	0.01–4 μg/L	[108]

Note: Gd_2_O_3_-MoS_2_@CNT NCs: Gadolinium oxide-MoS_2_ @carbon nanotube nanocomposites; Mn-MoS_2_/MWCNTs/NA/GCE: Mn-doped MoS_2_/MWCNTs/Nafion-modified glassy carbon electrode; PEI-MoS_2_@Au NPs: Polyethylenimine-functionalized MoS_2_@Au Nanoparticles;1T-MoS_2_/PANI/GCE: Polyaniline-functionalized 1T-MoS_2_ modified Glassy Carbon Electrode; SG/CNT-COOH/MoS_2_/Yeast: Sulfur-Doped Graphene/Carboxylated Carbon Nanotube/MoS_2_/Yeast Nanocomposite; MSQD/PGE: MoS_2_ Quantum Dots modified Pencil Graphite Electrode; Fe_3_O_4_/MoS_2_/Nafion/GCE: Nano-Fe_3_O_4_/MoS_2_/Nafion modified Glassy Carbon Electrode; rGO/MoS_2_/CS/GCE: Reduced Graphene Oxide/MoS_2_/Chitosan modified Glassy Carbon Electrode; CS/GO/MoS_2_/AuNPs: Chitosan/Graphene Oxide/MoS_2_/Gold Nanoparticles.

## 5. Challenges and Prospects

Despite the demonstrated advantages of MoS_2_-based composites in electrochemical heavy metal detection, their practical implementation faces multifaceted challenges.

- Synthesis Limitations: The preparation methodology critically governs MoS_2_’s crystalline structure, specific surface area, and defect density—parameters dictating electron transfer efficiency, active site distribution, and ultimately sensing performance. Top-down strategies (e.g., mechanical/liquid-phase exfoliation) yield structurally intact few-layer MoS_2_ with minimal defects but suffer from low throughput and inconsistent layer uniformity, hindering scalability. Conversely, bottom-up approaches (hydrothermal synthesis, chemical vapor deposition) enable mass production yet require stringent parameter control; suboptimal conditions induce excessive sulfur vacancies and lattice defects that degrade conductivity and electrocatalytic activity. These processes also pose intrinsic safety hazards due to high-temperature/pressure requirements.
- Material Design Trade-offs: Engineering composite architecture necessitates balancing competing properties. Noble metal nanoparticles offer exceptional electroactivity but incur prohibitively high costs. Conducting polymers exhibit limited electrochemical stability. Carbon matrices require complex surface functionalization. Metal oxides suffer from inherent poor conductivity. Biomaterials demonstrate inadequate long-term stability. Systematic optimization of electroactivity, conductivity, durability, and biocompatibility remains imperative.
- Real-World Application Barriers: Complex matrices (e.g., hypersaline wastewater, biofluids) introduce interfering species that compromise selectivity and accuracy. Kinetically inefficient preconcentration of trace metal ions at electrode surfaces fundamentally restricts sensitivity and analysis speed. For flexible devices, achieving mechanical robustness and batch-to-batch reproducibility presents additional hurdles to reliability and scalability. Furthermore, the transition from lab-scale fabrication (e.g., manual drop-casting) to industrial-scale manufacturing remains a significant challenge, as it requires overcoming issues of poor reproducibility and low throughput to ensure consistent sensor performance.

Future research must focus on addressing the challenges to advance the practical application of MoS_2_-based electrode materials in the electrochemical detection of heavy metals. In terms of synthesis, the development of low-cost, highly controllable, and scalable production methods is paramount, such as exploring continuous-flow microreactor synthesis or green processes to overcome the limitations of producing toxic by-products in traditional chemical vapor deposition and poor controllability in hydrothermal methods. Developing anti-fouling strategies, such as constructing protective coatings or self-cleaning interfaces, is crucial to ensure long-term stability and accuracy in real-world applications. In terms of scalable production, innovative manufacturing strategies such as automated inkjet printing and screen-printing should be leveraged to replace manual processes, enabling the high-throughput and reproducible fabrication of sensors. To mitigate ion interference in complex samples, novel matrix isolation techniques combined with highly selective recognition strategies should be employed, leveraging steric hindrance effects and specific binding mechanisms to block competitive adsorption by coexisting ions, thereby enhancing detection specificity. For accurate quantification in complex food matrices, the standard addition method should be routinely employed to compensate for severe matrix effects, overcoming the limitation of unavailable matched calibration standards. At the materials design level, strategies for compositing MoS_2_ with functional materials require further refinement; heterostructure interface engineering should be optimized to improve electron transfer efficiency. Concurrently, defect engineering, phase engineering, and strain engineering offer pathways to construct high-density active sites. Integrating these approaches with strategies to enhance interfacial charge transfer kinetics can shorten the adsorption equilibrium time for trace HMIs, consequently improving sensor sensitivity and response speed. For flexible device development, emphasis should be placed on designing composite materials that simultaneously exhibit high mechanical stability and good flexibility. Batch production processes must also be optimized to ensure device consistency and environmental adaptability. Furthermore, integrating the development of portable detection equipment will enable on-site rapid testing and real-time monitoring, providing robust technical support for environmental monitoring, food safety, and biomedical applications. Future breakthroughs will depend on deep interdisciplinary integration: in situ characterization techniques will be crucial to elucidate interfacial reaction kinetics, while machine learning algorithms can establish structure–property relationship models linking synthesis parameters to material structure and performance characteristics. This integrated approach will form a closed-loop innovation system from material design to end-user application, accelerating the widespread deployment of MoS_2_-based sensors in complex real-world scenarios. In summary, the thorough exploration and innovation along these research directions hold significant potential for substantially advancing the performance and application scope of MoS_2_-based electrode materials in heavy metal electrochemical detection.

## 6. Conclusions

MoS_2_-based composites demonstrate considerable promise for electrochemical heavy metal ion detection. This comprehensive review systematically examines the crystalline characteristics, synthesis methodologies, and structure–property relationships governing MoS_2_’s sensing performance, while critically evaluating recent application advances and persistent challenges. The material’s distinctive lamellar architecture, tunable electronic bandgap, and abundant catalytically active edge sites collectively enhance detection sensitivity and selectivity when utilized as electrode modifiers. Integration with metal nanoparticles, conductive polymers, carbon matrices, metal oxides, and biomacromolecules synergistically optimizes interfacial charge transfer, electrocatalytic activity, and target preconcentration—enabling ultralow detection limits and broad dynamic linear ranges. Nevertheless, practical implementation faces critical barriers, including scalable synthesis with precise defect control, matrix interference in complex samples, kinetically sluggish trace metal preconcentration, and long-term stability constraints in flexible devices. Future research must pioneer cost-effective synthesis routes while integrating novel matrix-separation technologies with selective recognition strategies, alongside rational heterocomposite engineering via interfacial design. Multidisciplinary convergence will ultimately accelerate the translational deployment of MoS_2_-based electrochemical sensors across environmental monitoring, food safety surveillance, and biomedical diagnostic applications.

## Figures and Tables

**Table 1:** Advantages and disadvantages of common synthesis techniques for MoS_2_.

Synthetic Method		Advantages	Disadvantages
Top-down approaches	Mechanical exfoliation	High quality, simple operation	Uncontrollable size, number of layers
	Chemical exfoliation	High quality, high efficiency	Complex operation, impurities
	Electrochemical exfoliation	High quality, low cost	Complex operation, uncontrollable number of layers
	Liquid-phase exfoliation	High quality, easy to operate	Low single-layer yield, uncontrollable number of layers
Bottom-up approaches	hydrothermal method	High quality, easy to operate	Long reaction time, higher reaction temperature
	Chemical vapor deposition	High quality, large area	Toxic by-products, higher reaction temperature

## Data Availability

No new data were created or analyzed in this study.
